# Optimization of Bioethanol Production Using Whole Plant of Water Hyacinth as Substrate in Simultaneous Saccharification and Fermentation Process

**DOI:** 10.3389/fmicb.2015.01411

**Published:** 2016-01-07

**Authors:** Qiuzhuo Zhang, Chen Weng, Huiqin Huang, Varenyam Achal, Duanchao Wang

**Affiliations:** Shanghai Key Lab for Urban Ecological Processes and Eco-Restoration, School of Ecological and Environmental Sciences, East China Normal UniversityShanghai, China

**Keywords:** water hyacinth, bioethanol, RSM, SSF, optimization methods

## Abstract

Water hyacinth was used as substrate for bioethanol production in the present study. Combination of acid pretreatment and enzymatic hydrolysis was the most effective process for sugar production that resulted in the production of 402.93 mg reducing sugar at optimal condition. A regression model was built to optimize the fermentation factors according to response surface method in saccharification and fermentation (SSF) process. The optimized condition for ethanol production by SSF process was fermented at 38.87°C in 81.87 h when inoculated with 6.11 ml yeast, where 1.291 g/L bioethanol was produced. Meanwhile, 1.289 g/L ethanol was produced during experimentation, which showed reliability of presented regression model in this research. The optimization method discussed in the present study leading to relatively high bioethanol production could provide a promising way for Alien Invasive Species with high cellulose content.

## Introduction

The major source of energy comes from non-renewable fossil fuel that caused global warming, environmental degradation, and human health problems (Patil et al., 2014). The growing energy demands encourage scientists to explore low cost, environmental friendly and sustainable alternative energy sources (Cheng et al., 2015a,b; Lin et al., 2015; Merino-Pérez et al., 2015).

Bioethanol, as a clean, safe and renewable resource, is considered as a potential alternative to fossil fuels (Rezania et al., 2015). However, it is mainly produced from either starch- or sugar-rich crops that may raise land competition between food production and biomass energy utilization, and can lead to deforestation (Zhao and Xia, 2010; Das et al., 2015). Hence, lignocellulose is gradually considered as more attractive because of its low cost and easy availability (Valentine et al., 2012; Bayrakci and Koçar, 2014).

Water hyacinth *(Eichornia crassipes)*, which is originated from Amazon basin (Barrett, 1989), is listed as one of the world's most invasive and recalcitrant weeds because of its availability in large quantities, extraordinary adaptive ability, and remarkable growth rate (Hu et al., 2015). It grows at an extreme rapid rate and produce almost 2 tons of biomass per acre and its population doubles every 5–15 days (Craft et al., 2003). Water hyacinth is usually blamed for depleting nutrients and oxygen from water bodies, increasing evapotranspiration, and reducing biodiversity, which could influence fishing, shipping, irrigation, and destroy aquatic eco-system (Malik, 2007; Guerrero-Coronilla et al., 2015). This species was brought to mainland China in the 1930s. With its extremely high growth rate, this floating plant has infested many aquatic systems in 19 provinces of China (Xia et al., 2013). Moreover, water hyacinth is considered as an attractive raw material for the bioenergy production including bioethanol, hydrogen, and biochar in many tropical regions of the world among various types of lignocellulosic substances (Masto et al., 2013; Buller et al., 2015; Jiu et al., 2015; Zhang et al., 2015).

Due to its abundant availability and high carbohydrate contents, water hyacinth highly satisfies the requirements as a potential substrate for bioethanol production (Ganguly et al., 2012; Rezania et al., 2015). The dry biomass of water hyacinth mainly comprises low lignin (7–26%) and high amount of cellulose (18–31%) and hemicellulose (18–43%), which can be easily hydrolyzed to reducing sugars and then fermented to bioethanol by effective yeasts (Bergier et al., 2012). However, there are many problems hindering the effective enzymatic hydrolysis. One of these problems is the lignin seal that prevents penetration by degrading enzymes (Taniguichi et al., 2005). Thus, many researchers tried to seek effective pretreatment methods to break the lignin seal (Forrest et al., 2010; Ma et al., 2010; Gao et al., 2013; Yan et al., 2015). Another bottleneck is the feedback inhibition of cellobiose on fermentation process after hydrolysis during bioethanol production (Guan et al., 2013; Ha et al., 2013; Cheng et al., 2015a,b). The most effective method to solve the feedback inhibition problem is simultaneous saccharification and fermentation (SSF), a process in which enzymatic process hydrolyzes lignocelluloses to sugars and ferments to bioethanol simultaneously, being already used in many lignocelluloses fermentation systems (Huang et al., 2013; Soares and Gouveia, 2013). However, there is no detail report on SSF process using water hyacinth as substrate.

In the present study, water hyacinth was collected from wastewater and pretreated. The major factors affecting the efficiency of SSF process were analyzed by response surface method (RSM) that provided an optimal fermentation parameter for bioethanol production by water hyacinth.

## Materials and methods

### Materials and microorganism

Water hyacinth was obtained from Huangpu River, Minhang District, Shanghai, China. It was washed three times with tap water to remove extraneous matter and roots, and then smashed by grinder below 40 meshes for further use.

Cellulase extracted from *Trichoderma viride* was bought from Sinopharm Chemical Reagent Co., Ltd, Shanghai. The activity of cellulase is 15000 U/g.

*Saccharomyces cerevisiae* was preserved in Shanghai Key Lab for Urban Ecological Processes and Eco-Restoration at East China Normal University (SHUES, ECNU), Shanghai. To prepare the yeast inoculum, one loop of *S. cerevisiae* spores was suspended in fluid enrichment medium (10 g/L yeast extract, 20 g/L peptone and 20 g/L glucose) for 24 h. The exponential inoculum was centrifuged at 4000 r/min for 5 min, followed by washing the precipitate by deionized water. After calculating dry weight of the yeast, 2 g/L yeast inoculum was prepared finally.

### Pretreatment of water hyacinth

Smashed water hyacinth was pretreated before enzymatic hydrolysis. Acid pretreatment (1% H_2_SO_4_ at 100°C for 30 min when solid-liquid ratio was 1:30), alkaline pretreatment (0.5% NaOH at 40°C for 30 min when solid-liquid ratio was 1:16) and microwave-alkaline combined pretreatment (150 W microwave combined with 0.5% NaOH for 0.5 min when solid-liquid ratio was 1:16) were used after optimization by orthogonal experiment. The specific conditions of the orthogonal experiment followed our previous research (Wang, 2015). Reducing sugars in hydrolysates were also determined.

### Enzymatic hydrolysis

The solid residue after pretreatment was collected by filtration and washed extensively with distilled water until neutral pH. Subsequently, this pretreated water hyacinth was dried in the oven at 70°C to maintain a constant weight to be used as the substrate for enzymatic hydrolysis.

Cellulase dosage, hydrolysis temperature and time were selected as three factors for single factor experiment. The reducing sugars in hydrolysates were detected to determine an optimum enzymatic hydrolysis process.

### Saccharification and fermentation process

One gram smashed water hyacinth sample was firstly pretreated by acid (1% H_2_SO_4_) at the optimal condition followed by alkali (6 mol/L NaOH) was used to regulate pH value to 5.3. After that the mixture was autoclaved at 121°C for 20 min, and 0.05 g cellulase with 0.05 g CaCl_2_ were added to the pretreated sample to hydrolyze. Meanwhile, yeast inoculum was added into pretreated sample according to the experimental design along with 2.0 g/L yeast extract, 0.2 g/L (NH_4_)_2_HPO_4_ and 0.02 g/L MgSO_4_. Nitrogen was aerated to exclude air in the system, and SSF was carried out at constant temperature shaker with the speed of 120 rpm. After SSF process, the fermented samples were centrifuged at 5000 rpm for 8 min and supernatant was used for bioethanol determination.

### Response surface method

The main factors that affect ethanol production, including fermentation temperature (X_1_), fermentation time (X_2_) and inoculums dosage (X_3_), were chosen to be optimized by RSM. Seventeen group experiments designed by Box-Behnken were conducted to seek the highest ethanol production, which were listed in **Table 2**.

### Analytical methods

The moisture content of water hyacinth was detected by oven drying method at 105°C for three times, and the average value was calculated ultimately. Cellulose, hemicelluloses and lignin contents in water hyacinth were determined by the methods of Goering and Van soest (1970). The DNS method was used for the measurement of total reducing sugar contents (Miller, 1959).

The bioethanol production during fermentation was measured using headspace sampling Gas Chromatography (GC, Agilent 7890A) method. HS-9A was selected as heated static headspace. The treated samples were examined by GC on a HP-5MS capillary column (30 m × 0.25 mm × 0.25 μm) with FID detector. Nitrogen was used as carrier gas. All the experiments and sample injection were performed in triplicates, and the average values were represented and used ultimately. The conditions were as follow:

(1) Static headspace: Vial 70°C; TR Line 90°C; Loop 80°C, 1 mL; Loop Fill Time 1 min; Ressuriz Time 0.2 min; Vial EQ Time 10 min.

(2) GC: The inlet temperature for GC was kept at 180°C with a Split ratio of 30:1. The oven temperature was kept at 180°C for 5 min at a flow rate of 2 mL-min^−1^.

(3) FID: Temperature 80°C; Hydrogen flow 30 mL-min^−1^; Air flow 400 mL-min^−1^; Make-up gas flow 25 mL-min^−1^.

## Results

### Constitution of water hyacinth

The average value of moisture content in water hyacinth sample was 90.85%, which is much higher than the other cellulosic wastes. The contents of cellulose, hemicellulose and lignin in different organs of water hyacinth were determined (Table 1). Compared to leaf and stem, the whole plant of water hyacinth possessed a little higher cellulose (18.07%) and hemicelluloses content (28.21%), and low lignin content (7.03%). Part of the erosion roots that was included in the whole plant of water hyacinth was removed before hydrolysis, so the composition of roots was not provided in Table 1. Considering the convenience and validity of utilization, the whole plant was finally selected as substrate for bioethanol production.

**Table 1:** **Constitution of water hyacinth in different organs**.

	**Contents (%)**			
**Organs**	**Cellulose**	**Hemicellulose**	**Lignin**	**Others**
Leaf	15.42 ± 0.08	29.75 ± 0.15	9.79 ± 0.06	45.04 ± 0.29
Stem	17.14 ± 0.12	21.82 ± 0.06	8.01 ± 0.07	53.03 ± 0.25
Whole plant	18.07 ± 0.20	28.21 ± 0.11	7.03 ± 0.09	46.69 ± 0.40

### Pretreatment and hydrolysis of water hyacinth

Three kinds of pretreatment methods, including acid pretreatment, alkali pretreatment and microwave-alkaline combined pretreatment, were used before hydrolysis and fermentation. Acid pretreatment was the most effective method to increase the sugar contents in samples. After grinding to 60 mesh, the water hyacinth sample was pretreated by 1% sulfuric acid at 100°C for 30 min when solid-liquid ratio was 1:30. An amount of 197.60 mg/g reducing sugars was obtained in hydrolysates. However, only 22.41 mg/g and 99.12 mg/g reducing sugars were obtained after alkaline pretreatment and microwave-alkaline combined pretreatment, respectively. Calculated by Hu's method (Hu and Wen, 2008), 37.9, 4.30, and 19.01% water hyacinth samples were converted into reducing sugars after acid pretreatment, alkaline pretreatment and microwave-alkaline combined pretreatment, respectively.

**Figure 1:**
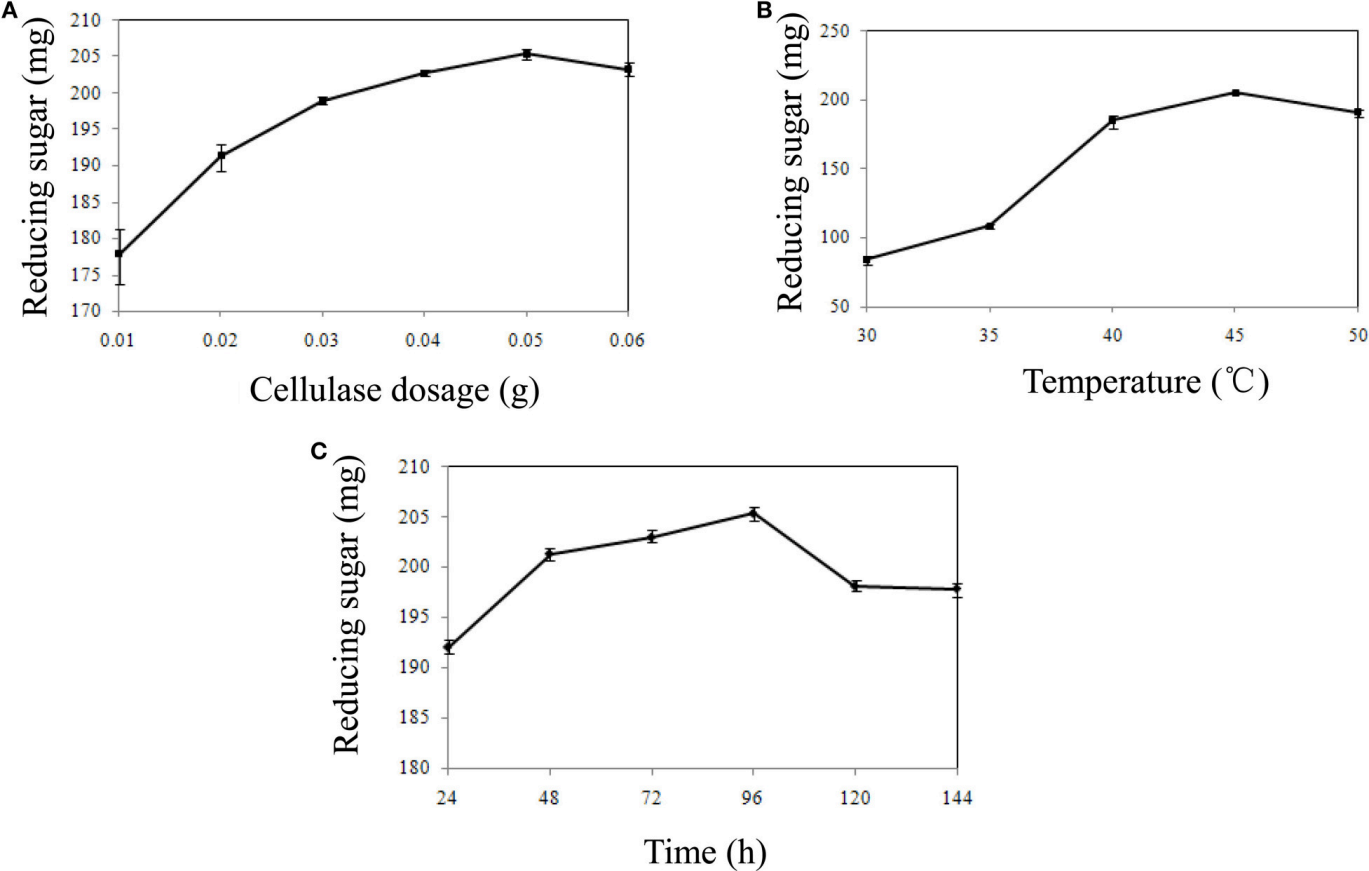
**The influence of hydrolysis factors on reducing sugar production**. **(A)** The influence of cellulase dosage on reducing sugar production. **(B)** The influence of temperature on reducing sugar production. **(C)** The influence of time on reducing sugar production.

The surplus residue part of water hyacinth was hydrolyzed by cellulase after pretreatment. Various factors that influence cellulase hydrolysis, including cellulase dosage, enzyme temperature and reaction time, were optimized by single factor experiment (Figures 1A–C). The result showed that the yield of reducing sugar could reach 205.33 mg at 45°C at the end of 96 h when 0.05 g cellulase was added. Thus, totally 402.93 mg (197.60 mg in hydrolysates and 205.33 mg by residue hydrolysis) reducing sugar could be produced at the optimal condition.

### Regression model and significance test by response surface method in simultaneous saccharification and fermentation process

The Box-Behnken design and experimental results were listed in Table 2. Variance test and regression were analyzed by Design-expert software after manual operation, which was shown in Table 3. It was showed that the regression model was highly significant (*P* < 0.05) and the regression equation obtained in the present study could predict the response value effectively.

**Table 2:** **Box-Behnken design and experimental results**.

	**Factor**			
**No**.	**X_1_**	**X_2_**	**X_3_**	**Bioethanol concentration (g/L)**
1	−1	−1	0	1.07
2	1	−1	0	0.66
3	−1	1	0	0.56
4	1	1	0	1.11
5	−1	0	−1	0.49
6	1	0	−1	0.93
7	−1	0	1	0.43
8	1	0	1	1.02
9	0	−1	−1	0.63
10	0	1	−1	0.65
11	0	−1	1	0.65
12	0	1	1	0.68
13	0	0	0	1.14
14	0	0	0	1.28
15	0	0	0	1.21
16	0	0	0	1.31
17	0	0	0	1.23

**Table 3:** **Variance analysis of regression equation**.

**Source**	**Sum of squares**	**df**	**Mean square**	***F*-value**	***P*-value**
Model	1.34	7	0.19	13.99	0.0004
X_1_	0.17	1	0.17	12.47	0.0064
X_2_	1.250E-005	1	1.250E-005	9.106E-004	0.9766
X_3_	8.000E-004	1	8.000E-004	0.058	0.8146
X_1_X_2_	0.23	1	0.23	16.78	0.0027
X_1_^2^	0.11	1	0.11	7.80	0.0209
X_2_^2^	0.21	1	0.21	15.46	0.0034
X_3_^2^	0.54	1	0.54	39.09	0.0001
Residual	0.12	9	0.014	–	–
Lack of fit	0.11	5	0.021	4.91	0.0743
Pure error	0.017	4	4.330E-003	–	–
Cor total	1.47	16	–	–	–

The monomial expression X_1_ (*P* = 0.0064) was significant, whereas X_2_ (*P* = 0.9766) and X_3_ (*P* = 0.8146) were nonsignificant. Thus, fermentation temperature was the most crucial factor that could influence SSF process. The binomial expression X_3_^2^ (*P* = 0.0001) was extremely significant, X_2_^2^ (*P* = 0.0034) was relatively significant, and there was certain significance for X_1_^2^ (*P* = 0.0209). The results indicated the primacy sequence influencing SSF process as fermentation temperature > inoculums dosage > fermentation time.

The secondary multivariate regression equation obtained from Design-expert software was *Y* = 1.23+0.15X_1_ − (1.250E-003X_2_) + 0.010X_3_ + 0.24 X_1_X_2_ − 0.16 X_1_^2^ − 0.22 X_2_^2^ − 0.36 X_3_^2^. According to the secondary multivariate regression equation and regression model, the optimal condition for bioethanol production by SSF process was fermented at 38.87°C for 81.87 h when inoculated with 6.11 ml yeast. The ethanol yield could achieve 1.291 g/L at this condition by our regression model. Furthermore, 1.289 g/L ethanol was produced during real experimentation at this condition, which is very close to our regression model.

**Figure 2:**
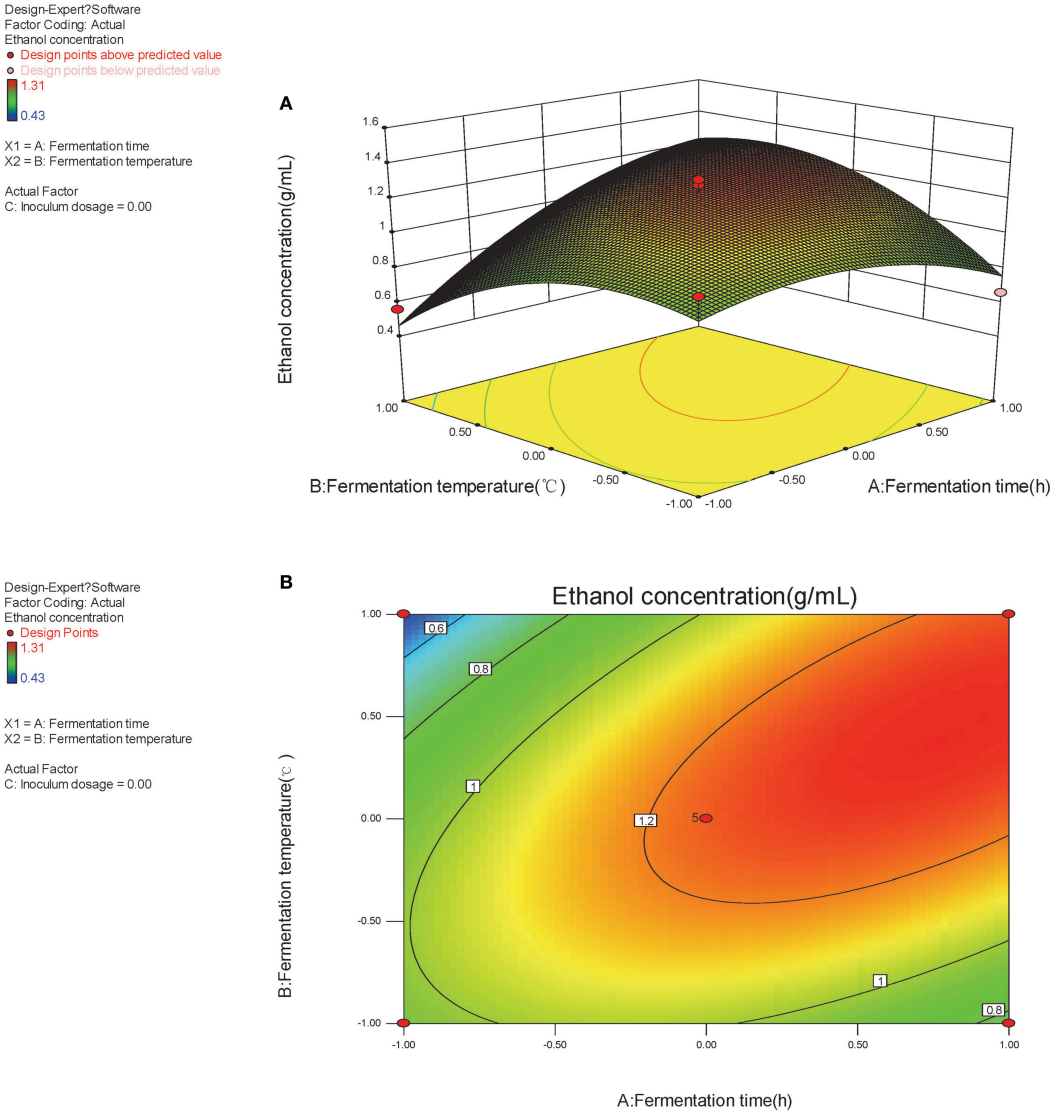
**(A,B)** Response of bioethanol production to fermentation time and fermentation temperature.

**Figure 3:**
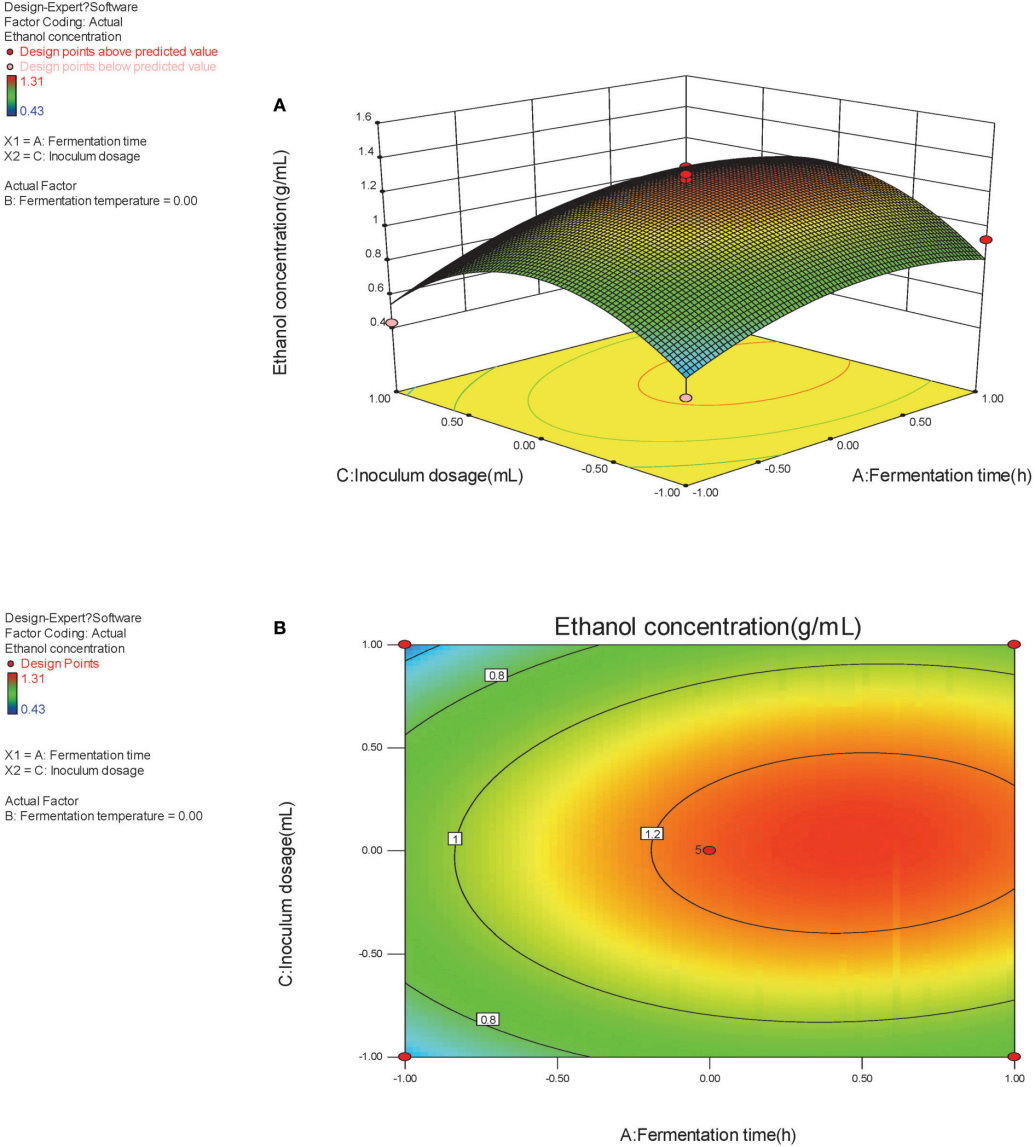
**(A,B)** Response of bioethanol production to fermentation time and inoculums dosage.

**Figure 4:**
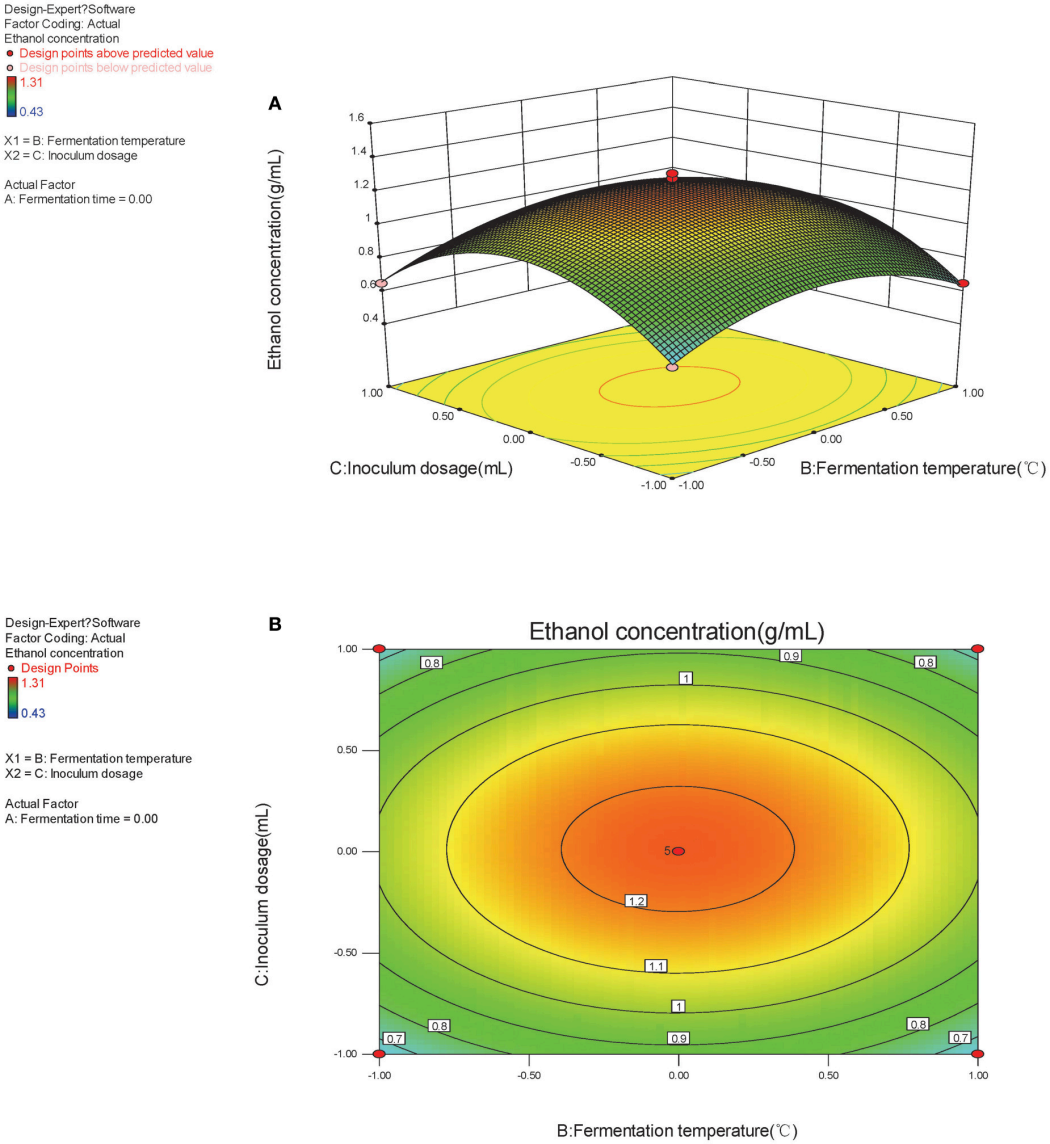
**(A,B)** Response of bioethanol production to fermentation temperature and inoculums dosage.

The optimal factors and factor levels could be determined and the interaction effects between two factors could be revealed by response surface and contour map. The response surface and contour map of the secondary multivariate regression equation was shown in Figures 2A,B, 3A,B, and 4A,B, respectively. The results showed a remarkable significant interaction between fermentation temperature (X_1_) and fermentation time (X_2_). However, there was no significant interaction between fermentation time (X_2_) and inoculums dosage (X_3_). Moreover, there was no significant interaction between fermentation temperature (X_1_) and inoculums dosage (X_3_), either. Fermentation temperature (X_1_) was the most important factor influencing SSF process in the present study.

## Discussion

Based on our results, the whole plant of water hyacinth possesses the highest cellulose content compared to stem and leaf after removing its rotten part. Therefore, it was selected as substrate for ethanol production, which could make full use of the whole plant.

Biofuel production from cellulosic materials greatly depends on the disruption of its complex lignocellulosic structure, which is recalcitrant to biodegradation. Hence, a suitable and effective pretreatment method should be selected for different cellulosic substrates. Various kinds of pretreatment methods, including acid, alkali, microwave, liquid hot water and compound pretreatments, were investigated nowadays for cellulose hydrolysis (Forrest et al., 2010; Ma et al., 2010; Gao et al., 2013; Timung et al., 2015; Yan et al., 2015). In our previous study, alkaline pretreatment was optimal for bioethanol production by rice straw hydrolysis (Zhang and Cai, 2008). However, acid pretreatment was the most effective method for bioethanol production by water hyacinth compared to other pretreatment methods in this study. The possible mechanism involves dissolution of hemicellulose causing loosening of the structure of raw material, which makes acid pretreatment an important method for the production ofreducing sugars (Ma et al., 2010; Xia et al., 2013). After pretreated by acid, the loosen substrate could increase the contact area between cellulose and cellulase. Meanwhile, it might prevent unproductive binding of enzymes to lignin, thus facilitates reducing sugar production (Zhang et al., 2009).

Cellulase usage is a huge cost in bioethanol production process. Large amounts of cellulases are needed to hasten the hydrolysis process, thereby increasing the cost of the whole processing. In addition, enzyme recycling becomes difficult because of the adsorption of cellulases to residual cellulosic materials (Zhang et al., 2009). Thus, many factors, such as cellulase dosage, enzyme temperature and reaction time, are urgently necessary to be optimized to reduce the cost of bioethanol production. After optimization of such important parameters in the present study, 402.93 mg (197.60 mg in hydrolysates, and 205.33 mg by residue hydrolysis) reducing sugar was produced. The theoretical yield of reducing sugar that produced by water hyacinth was 521.30 mg, counted by Hu's method (Hu and Wen, 2008). Therefore, the real yield of achieved reducing sugar was 77.29% of the theoretical yield by our optimized technique, and is more effective method compared to other reports, which only produce reducing sugars from the hydrolysates (Ma et al., 2010; Yan et al., 2015). However, pentose (such as xylose) sugar could not be digested using *S. cerevisiae* and remained unchanged during fermentation (Zhang and Cai, 2008). Hence, exploring a hexose-pentose co-fermentation system by genetic engineering method could be a promising way to improve bioethanol production by cellulosic materials.

According to our regression model, the ethanol yield could achieve 1.291 g/L at the optimum condition, while 1.289 g/L ethanol was produced by our real experiment at this condition, which is very close to the predicted model. These results showed the reliability of presented regression model. In terms of factors, which influenced SSF process, fermentation temperature (X_1_) was the most important compared to other factors, as temperature is crucial for growth and activity of cellulase and *S. cerevisiae.* Meanwhile, bioethanol production possessed a relatively high value that proved a fact that the SSF process is more effective than SSF separately (Wang et al., 2013; Asada et al., 2015; Saha et al., 2015). Although SSF could solve the feedback inhibition problem of cellobiose on fermentation process, it has some drawbacks, such as gap for the optimum temperature and pH conditions between cellulase and *S. cerevisiae.* Thus, a pivotal research field is required to develop and regulate a suitable condition for microorganisms in SSF process.

In addition, compared to other cellulosic materials, the moisture content in water hyacinth is much higher that could be solved during bioethanol production by fermentation of water hyacinth in future (Mishima et al., 2008). Further, there are some inhibitors (furfural, furan, phenols, etc.) in the hydrolysates that could destroy the fermentation process (Kalyani et al., 2012; Moreno et al., 2012; Wang et al., 2014), and an effective method to minimize the inhibitors production is urgently required in the future study. Moreover, the solid loading in enzymatic hydrolysis and SSF process might be another important factor, which need to be optimized in our future work.

## Conclusion

As a conventional invasive weed, water hyacinth proved its feasibility for bioethanol energy production. In our experiment, 402.93 mg reducing sugar and 1.289 g/L bioethanol were achieved using water hyacinth substrate in Simultaneous SSF process after being optimized by RSM. This relatively high bioethanol production indicates that water hyacinth is a promising plant in research and development of sustainable energy.

### Conflict of interest statement

The authors declare that the research was conducted in the absence of any commercial or financial relationships that could be construed as a potential conflict of interest.
